# Intraperitoneal injection of class A TLR9 agonist enhances anti–PD-1 immunotherapy in colorectal peritoneal metastases

**DOI:** 10.1172/jci.insight.160063

**Published:** 2022-10-24

**Authors:** Ting Jiang, Hongji Zhang, Yiming Li, Preethi Jayakumar, Hong Liao, Hai Huang, Timothy R. Billiar, Meihong Deng

**Affiliations:** 1 Department of Surgery, University of Pittsburgh, Pittsburgh, Pennsylvania, USA.; 2 Tsinghua University School of Medicine, Beijing, China.; 3 Department of Surgery, The Ohio State University, Columbus, Ohio, USA.; 4 Department of Surgery, University of Virginia, Charlottesville, Virginia, USA.; 5 Center for Immunology and Inflammation, Feinstein Institutes for Medical Research, Manhasset, New York, USA.; 6 Department of Microbial Infection and Immunity, The Ohio State University, Columbus, Ohio, USA.

**Keywords:** Immunology, Oncology, Cancer immunotherapy, Macrophages

## Abstract

Peritoneal metastases are associated with a low response rate to immune checkpoint blockade (ICB) therapy. The numbers of peritoneal resident macrophages (PRMs) are reversely correlated with the response rate to ICB therapy. We have previously shown that TLR9 in fibroblastic reticular cells (FRCs) plays a critical role in regulating peritoneal immune cell recruitment. However, the role of TLR9 in FRCs in regulating PRMs is unclear. Here, we demonstrated that the class A TLR9 agonist, ODN1585, markedly enhanced the efficacy of anti–PD-1 therapy in mouse models of colorectal peritoneal metastases. ODN1585 injected i.p. reduced the numbers of Tim4^+^ PRMs and enhanced CD8^+^ T cell antitumor immunity. Mechanistically, treatment of ODN1585 suppressed the expression of genes required for retinoid metabolism in FRCs, and this was associated with reduced expression of the PRM lineage–defining transcription factor GATA6. Selective deletion of TLR9 in FRCs diminished the benefit of ODN1585 in anti–PD-1 therapy in reducing peritoneal metastases. The crosstalk between PRMs and FRCs may be utilized to develop new strategies to improve the efficacy of ICB therapy for peritoneal metastases.

## Graphical abstract

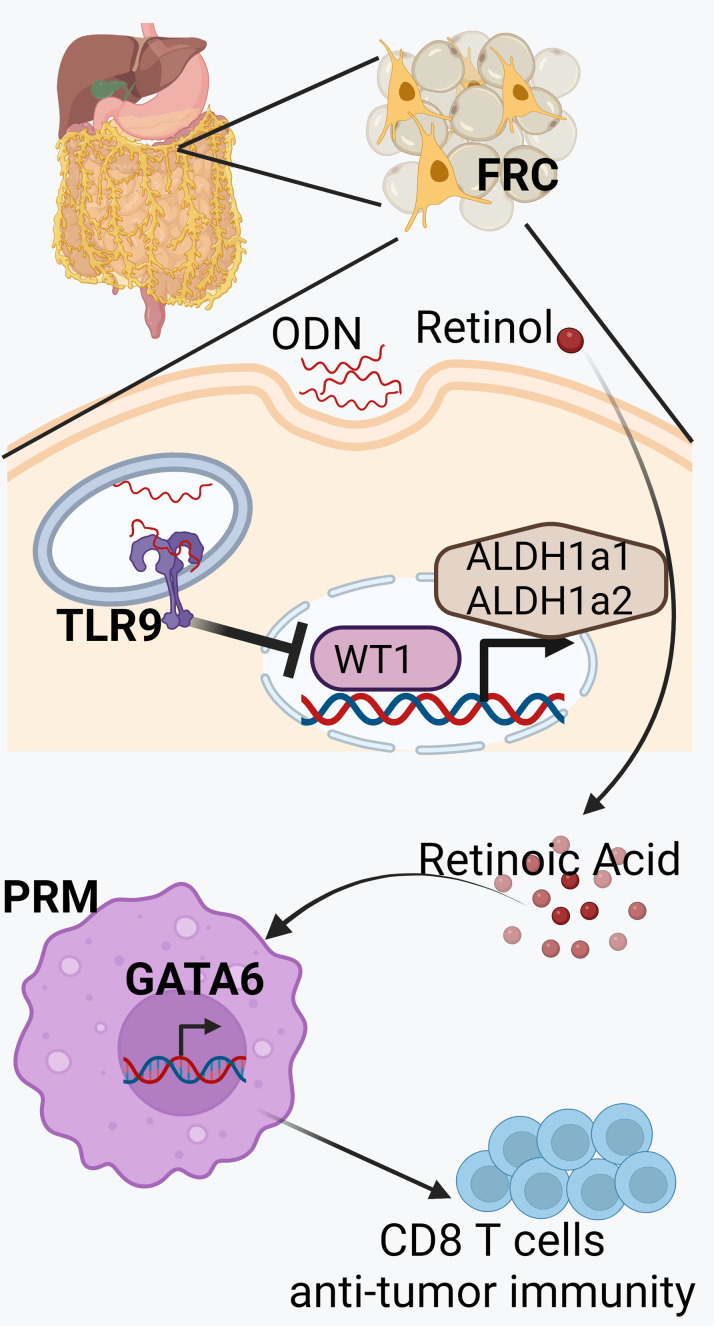

## Introduction

The peritoneal cavity is a common metastatic site of a variety of malignant diseases, including colorectal cancer (1), gastric cancer (2), ovarian cancer (3, 4), and lung cancer (5). Although various approaches, such as extended resections, combination chemotherapy, i.p. chemotherapy, and immunotherapy, have been attempted, the prognosis of patients with peritoneal metastases remains poor, and there is no established standard treatment (6). Immune checkpoint blockade (ICB) has shown promising therapeutic efficacy in cancer therapy; however, it has been shown that peritoneal metastases are associated with a low response rate to ICB and poor clinical outcomes after ICB therapy (5). Therefore, there is a need for novel immunotherapeutic strategies to improve the response rate of ICB therapy for peritoneal metastases.

The peritoneal immune environment regulates the progression of metastases and the response to ICB therapy (7). Peritoneal resident macrophages (PRMs) are a population of resident immune cells that play central roles in modulating the peritoneal immunity for tissue repair (8–11), pathogen clearance (12, 13), and tumor metastasis (2–5). Emerging evidence indicates that PRMs promote peritoneal metastasis in gastric (2), ovarian (3, 4), and lung cancer (5). The number of PRMs has been reported to reversely correlate to the prognosis of patients with gastric cancer metastatic to the peritoneum (2). Specific depletion of CD163^+^Tim4^+^ tissue-resident macrophages in the peritoneal cavity prevents the metastatic spread of ovarian cancer in mice (3). Furthermore, blocking Tim-4 on PRMs enhances the efficacy of anti–PD-1 ICB therapy in mouse models of colorectal peritoneal metastases (5). These data suggest that controlling the number of PRMs may represent a new therapeutic strategy to prevent metastasis and enhance the efficacy of ICB in peritoneal metastases.

TLR9 is an endosomal receptor that senses endogenous or exogenous unmethylated CpG DNA in order to drive immune responses (14). Recent studies have shown that intratumoral injection of TLR9 agonists enhances the efficacy of anti–PD-1 ICB therapy in mouse models of pancreatic cancer (15) and head and neck squamous cell carcinoma (16). However, the effect of TLR9 agonists on the therapeutic efficacy of ant–PD-1 therapy in peritoneal metastases remains unknown. We have previously shown that activation of TLR9 signaling in fibroblastic reticular cells (FRCs), a unique subpopulation of stromal cells in fat-associated lymphoid clusters (FALCs) in the omentum and mesenteric adipose tissue, suppresses immune cell recruitment to the peritoneal cavity (17). However, the effect of exogenous TLR9 agonists on peritoneal tumor immunity is unknown.

In this study, we assessed the effect of exogenous TLR9 agonists on the efficacy of anti–PD-1 therapy in a mouse model of locoregional colorectal cancer peritoneal spread (18). Using the class A TLR9 agonist, ODN1585, and FRC-TLR9–selective knockout mice, we found an unexpected role for TLR9 in FRCs regulating PRM disappearance in colorectal peritoneal metastasis. ODN1585 suppressed the expression of genes required for retinoid metabolism in FRCs, which is known to drive GATA6 expression for PRM homeostasis (19, 20). These studies reveal the potential of class A TLR9 agonists that could be used to enhance the efficacy of PD-1–targeted therapies in treating peritoneal metastasis.

## Results

### ODN1585 improves the efficacy of anti–PD-1 therapy for colorectal peritoneal metastases.

**Figure 1:**
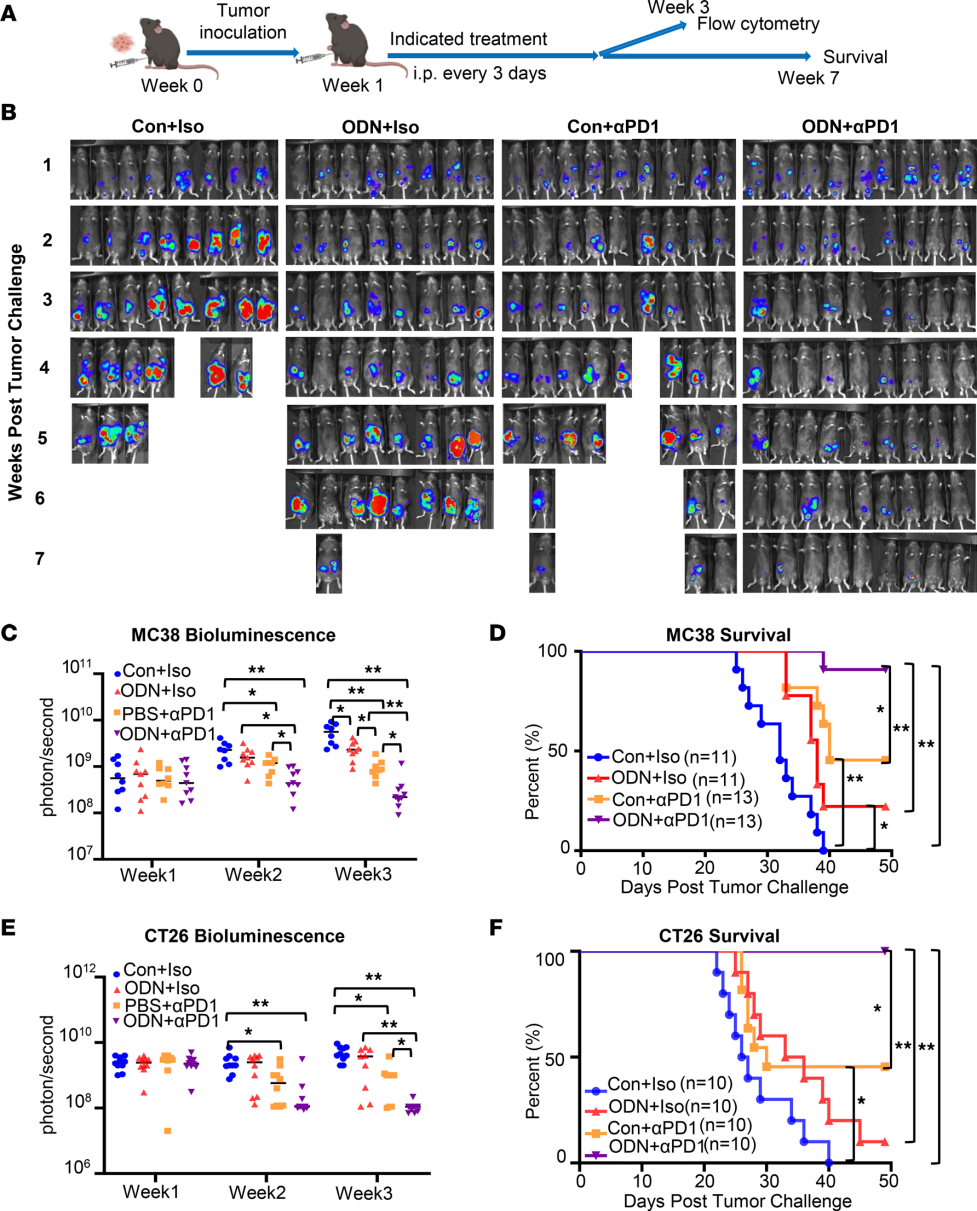
ODN1585 improves the efficacy of anti–PD-1 therapy for colorectal peritoneal metastases. (**A**) Schematic timeline of experimental setting and analysis for peritoneal tumor models. Mice received control and isotype antibodies (Con+Iso), ODN1585 (5 nmol/mouse) and Iso (ODN+Iso), control and anti–PD-1 (10 mg/kg, Con+αPD1), or ODN1585 and anti–PD-1 (ODN+αPD1) every 3days. (**B–D**) Mice were inoculated with MC38-luciferase-GFP (1 million cells/mouse, i.p.). (**B**) Bioluminescence images from individual WT mice at the indicated times after the peritoneal tumor challenge. One representative of 2 independent experiments is depicted. (**C**) Bioluminescence was measured as photons per second at the indicated times after the peritoneal tumor challenge. (**D**) Kaplan-Meier survival curve for 49 days. (**E** and **F**) BALB/cByJ mice were administered with CT26-luciferase-GFP cells i.p. (1 million cells/mouse). (**E**) Bioluminescence was measured as photons per second at the indicated times after peritoneal tumor challenge. (**F**) Kaplan-Meier survival curve. (**C** and **E**) Data are from 2 separate experiments. Symbols represent individual mice. Statistical differences were determined using a 2-way ANOVA with Tukey’s multiple comparisons test. (**D** and **F**) Data are from 2 separate experiments. Statistical differences were determined using log-rank test adjusted by Bonferroni’s testing. *P < 0.05, **P < 0.01. Only the significant differences are labeled.

Intratumoral injection of TLR9 agonists enhances the efficacy of anti–PD-1 ICB therapy in mouse models of pancreatic cancer (15) and head and neck squamous cell carcinoma (16). We have previously shown that TLR9 signaling plays a critical role in controlling the peritoneal immune environment (17). Therefore, we hypothesized that TLR9 agonists would enhance responses to anti–PD-1 therapy in peritoneal metastases. To test this hypothesis, we i.p. inoculated C57BL/6 WT mice with microsatellite-instable MC38 colorectal carcinoma cells (MC38-luciferase-GFP cells, 1 million/mouse, i.p.) to mimic locoregional colorectal cancer peritoneal spread. Mice were treated with control oligonucleotide and control isotype antibody, the class A TLR9 agonist ODN1585 (5 nmol/mouse, i.p.) and control isotype antibody, control isotype antibody and anti–PD-1 antibody, or ODN1585 and anti–PD-1 antibody (ODN+anti–PD-1) every 3 days starting 1 week after tumor inoculation (Figure 1A). Interestingly, treatment with ODN1585 reduced tumor burden in mice inoculated with MC38 murine colon carcinoma at week 3 and increased the 7-week-survival rate to about 25% compared with the 0% survival rate as seen in the control group (Figure 1, B–D). As expected, treatment of anti–PD-1 markedly reduced tumor burden starting as early as week 3 (Figure 1, B and C). Furthermore, the anti–PD-1 treatment improved the 7-week-survival rate to about 50% (Figure 1D). Notably, combined treatment with ODN+anti–PD-1 further decreased tumor burden at weeks 2 and 3 and increased the 7-week-survival rate to about 90% (Figure 1, B–D). Treatment with ODN1585 also substantially improved the therapeutic efficacy of anti–PD-1 therapy in BALB/cByJ mice inoculated with microsatellite-stable CT26 colon carcinoma cells (1 million cells/mouse, i.p.) (Figure 1, E and F). These data demonstrate that ODN1585 improves the efficacy of anti–PD-1 therapy in models of colorectal peritoneal metastases.

### ODN1585 alters peritoneal immunity during colorectal peritoneal metastases.

**Figure 2:**
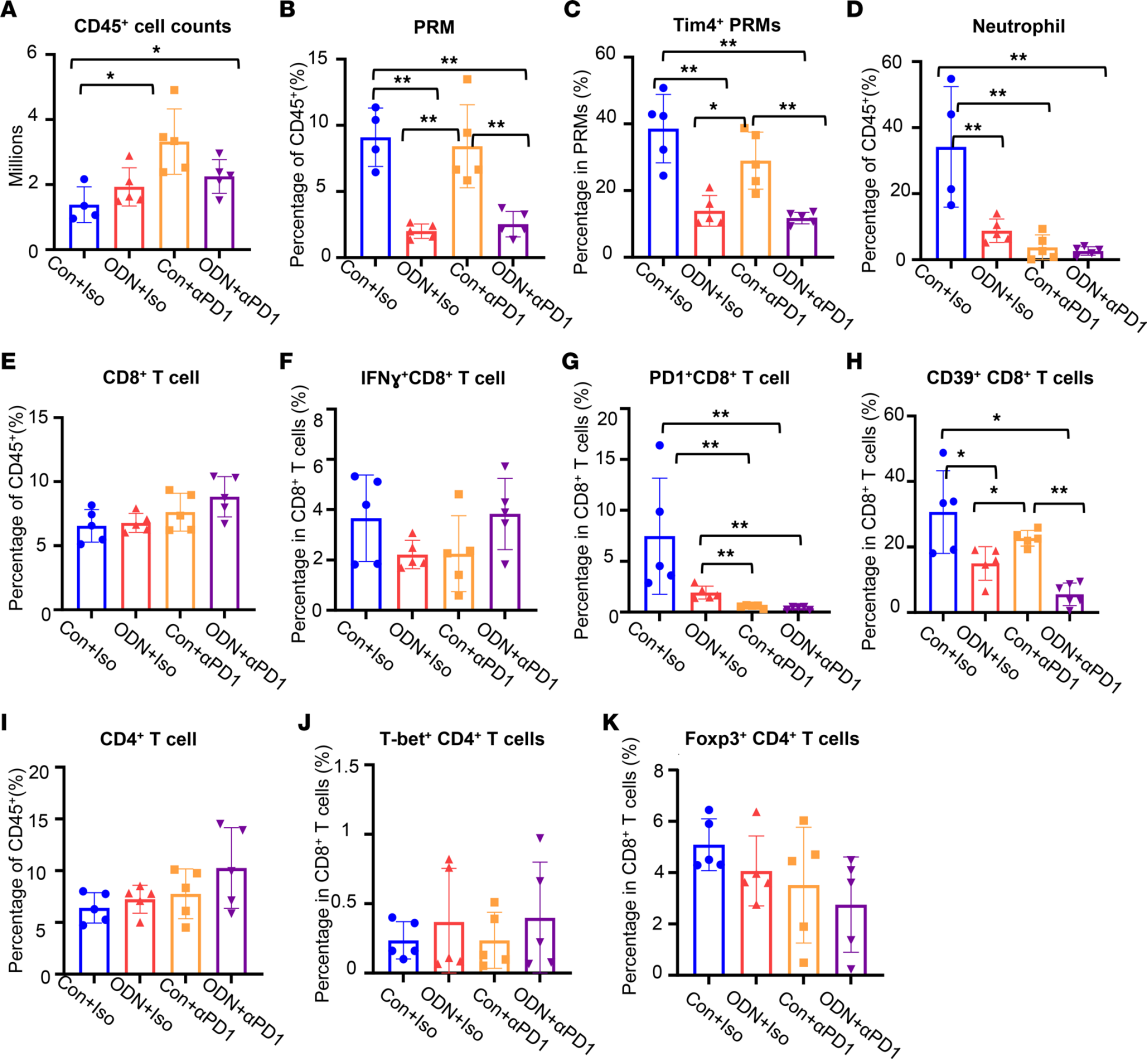
ODN1585 alters the peritoneal immunity during colorectal peritoneal metastases. Mice were inoculated with MC38-luciferase-GFP (1 million cells/mouse, i.p.) and received indicated treatment, as shown in Figure 1A. Peritoneal cells were collected at 3 weeks after tumor inoculation. The numbers and phenotypes of peritoneal cells were analyzed using flow cytometry. (**A**) The number of CD45^+^ cells. (**B**) The percentage of PRMs in CD45^+^ cells. (**C**) The percentage of Tim4^+^ cells in PRMs. (**D**) The percentage of neutrophils in CD45^+^ cells. (**E**) The percentage of CD8^+^ T cells in CD45^+^ cells. (**F–H**) The percentage of (**F**) IFN-γ^+^CD8^+^ T cells, (**G**) PD-1^+^CD8^+^ T cells, and (**H**) CD39^+^CD8^+^ T cells in CD8^+^ T cells. (**I**) The percentage of CD4^+^ T cells in CD45^+^ cells. (**J** and **K**) The percentage of (**J**) T-bet^+^CD4^+^ T cells and (**K**) Foxp3^+^CD4^+^ T cells in CD4^+^ T cells. Data are shown as mean ± SD from 2 separate experiments. Symbols represent individual mice. Statistical differences were determined using 1-way ANOVA with Tukey’s multiple comparisons test. *P < 0.05; **P < 0.01. Only the significant differences are labeled.

To test if treatment with ODN1585 alters the peritoneal immune environment during colorectal peritoneal metastases, we collected the peritoneal cells from mice at 3 weeks after MC38 tumor inoculation. The numbers and phenotypes of immune cells were analyzed using flow cytometry. The numbers of peritoneal CD45^+^ immune cells were substantially higher in mice that received anti–PD-1 treatment and ODN+anti–PD-1 treatment compared with those that received control treatment (Figure 2A). There was no significant difference in the numbers of peritoneal CD45^+^ immune cells between mice that received anti–PD-1 treatment and those that received ODN+anti–PD-1 treatment (Figure 2A). Interestingly, the percentage of PRMs and Tim4^+^ PRMs in CD45^+^ immune cells from mice that received ODN1585-only or ODN+anti–PD-1 treatment was substantially lower compared with that of mice that received control oligonucleotide and control isotype antibody or anti–PD-1–only treatment (Figure 2, B and C), suggesting that ODN1585 induces PRM disappearance from the peritoneal fluid. The percentage of neutrophils in CD45^+^ immune cells in mice that received ODN1585 only, anti–PD-1 only, and ODN+anti–PD-1 treatment was substantially lower than in mice that received the control treatment. Furthermore, the percentage of neutrophils was similar between mice that were given anti–PD-1 only and ODN+anti–PD-1 treatment (Figure 2D). These data indicate that the addition of ODN1585 to anti–PD-1 treatment decreases the numbers of PRMs and Tim4^+^ PRMs but not neutrophils compared with anti–PD-1–only treatment, suggesting that ODN1585 may enhance the efficiency of anti–PD-1 treatment for metastases via suppressing PRMs.

A recent study demonstrated that Tim4^+^ PRMs dampen the therapeutic efficacy of anti–PD-1 ICB by impairing antitumor immunity mediated by CD8^+^ T cells (5). We next tested whether ODN1585 altered T cell immunity after anti–PD-1 treatment. We first accessed the number and phenotype of CD8^+^ T cells in the peritoneum using flow cytometry. The percentage of CD8^+^ T cells and IFN-γ^+^CD8^+^ T cells was similar between treatments (Figure 2, E and F). Treatment with anti–PD-1 markedly decreased the percentages of PD-1^+^CD8^+^ T cells compared with treatment with control or ODN1585 only (Figure 2G). However, there was no significant difference in the percentage of PD-1^+^CD8^+^ T cells between mice that received anti–PD-1–only treatment or ODN+anti–PD-1 treatment (Figure 2G). Interestingly, we found that treatment with ODN1585 decreased the percentage of CD39^+^CD8^+^ T cells (Figure 2H), a subset of functionally exhausted CD8^+^ T cells (21). Notably, the percentage of CD39^+^CD8^+^ T cells in mice that received ODN+anti–PD-1 treatment was substantially lower than in mice that received anti–PD-1–only treatment (Figure 2H). In contrast, the percentage of CD4^+^ T cells, T-bet^+^CD4^+^ T cells, and Foxp3^+^CD4^+^ T cells was similar between treatments (Figure 2, I–K). These data suggested that the ODN1585 suppressed Tim4^+^ PRMs and, thus, enhanced CD8^+^ T cell antitumor immunity.

### Class A TLR9 agonists are potent inducers of PRM disappearance.

**Figure 3:**
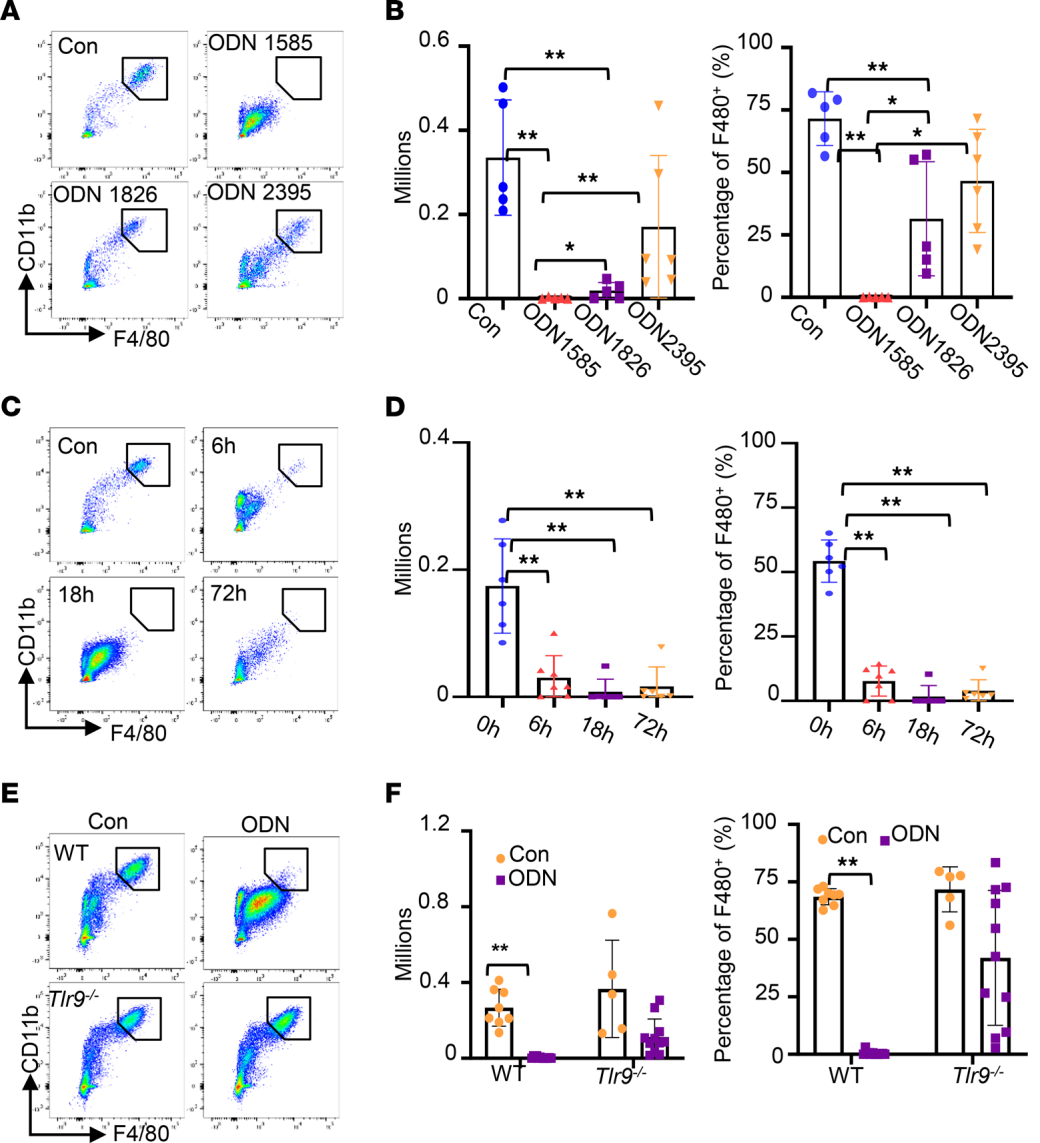
ODN1585 induced PRM disappearance in a TLR9-dependent manner. (**A** and **B**) WT mice were treated with PBS (100 μL, i.p.), ODN1585 (5 nmol/mouse, i.p.), ODN1826 (5 nmol/mouse, i.p.), or ODN2395, (5 nmol/mouse, i.p.) for 18 hours. (**A**) Representative gating of PRMs and PRM counts and (**B**) the percentage of PRMs in total F4/80^+^ cells are shown. (**C** and **D**) WT mice were treated with or without ODN1585 (5 nmol/mouse, i.p.) for 6, 18, and 72 hours. (**C**) Representative gating of PRMs and PRM counts and (**D**) the percentage of PRMs in total F4/80^+^ cells are shown. (**E** and **F**) WT and *Tlr9^–/–^* mice were treated with or without ODN1585 (5 nmol/mouse, i.p.) for 18 hours. (**E**) Representative gating of PRMs and PRM counts and (**F**) the percentage of PRMs in total F4/80^+^ cells are shown. Data are shown as mean ± SD from 2 separate experiments. Symbols represent individual mice. Statistical differences were determined using 1-way ANOVA with Tukey’s multiple comparisons test. *P < 0.05, **P < 0.01. Only the significant differences are labeled.

PRMs have been reported to disappear in the peritoneal fluid just hours after the initiation of inflammation in a phenomenon called the “macrophage disappearance reaction” (MDR). The above results indicated that the class A TLR9 agonist, ODN1585, induced PRM disappearance from the peritoneal fluid (13, 22). However, the mechanisms underlying the MDR are unclear. To test if other class A TLR9 agonists also induce PRM disappearance from the peritoneal fluid, WT mice were treated with 2 commercially available class A TLR9 agonists, ODN2216 and ODN2336 (5 nmol/mouse, i.p.), for 18 hours. We found that treatment with ODN2216 or ODN2336 dramatically reduced the numbers and percentages of PRMs to about 10% of the control treatment (Supplemental Figure 1; supplemental material available online with this article; https://doi.org/10.1172/jci.insight.160063DS1), suggesting that class A TLR9 agonists are potent inducers of PRM disappearance. There are 3 major classes of TLR9 agonists. Class A oligodeoxynucleotides, such as ODN1585, induce high IFN-α production from pDCs. Class B oligodeoxynucleotides, such as ODN1826, strongly activate B cells and TLR9-dependent NF-κB signaling. Class C oligodeoxynucleotides, such as ODN2395, combine features of classes A and B. To test the effect of different classes of oligodeoxynucleotides on PRM disappearance, WT mice were treated with ODN1585, ODN1826, or ODN2395 (5 nmol/mouse, i.p.) for 18 hours. Of note, PRMs almost completely disappeared from the peritoneal fluid after ODN1585 treatment (Figure 3, A and B). The number of PRMs dropped after ODN1826 treatment compared with the control treatment. However, a population of PRMs remained detectable (Figure 3, A and B). In contrast, the numbers of PRMs in ODN2395-treated mice were only slightly lower than in controls (Figure 3, A and B). Furthermore, the disappearance of PRMs from the peritoneal fluid was seen as early as 6 hours after ODN1585 treatment, and PRM numbers remained suppressed to 72 hours, the last time point assessed (Figure 3, C and D). Deletion of *Tlr9* mostly preserved total counts and frequency of PRMs in ODN1585-treated mice, as measured at 18 hours (Figure 3, E and F). These data indicated that class A TLR9 agonists are potent inducers of PRM disappearance in the peritoneal cavity. ODN1585 was used for the remainder of the experiments in this study.

### Activation of TLR9 in FRCs induced PRM disappearance via suppression of GATA6 expression in PRMs.

**Figure 4:**
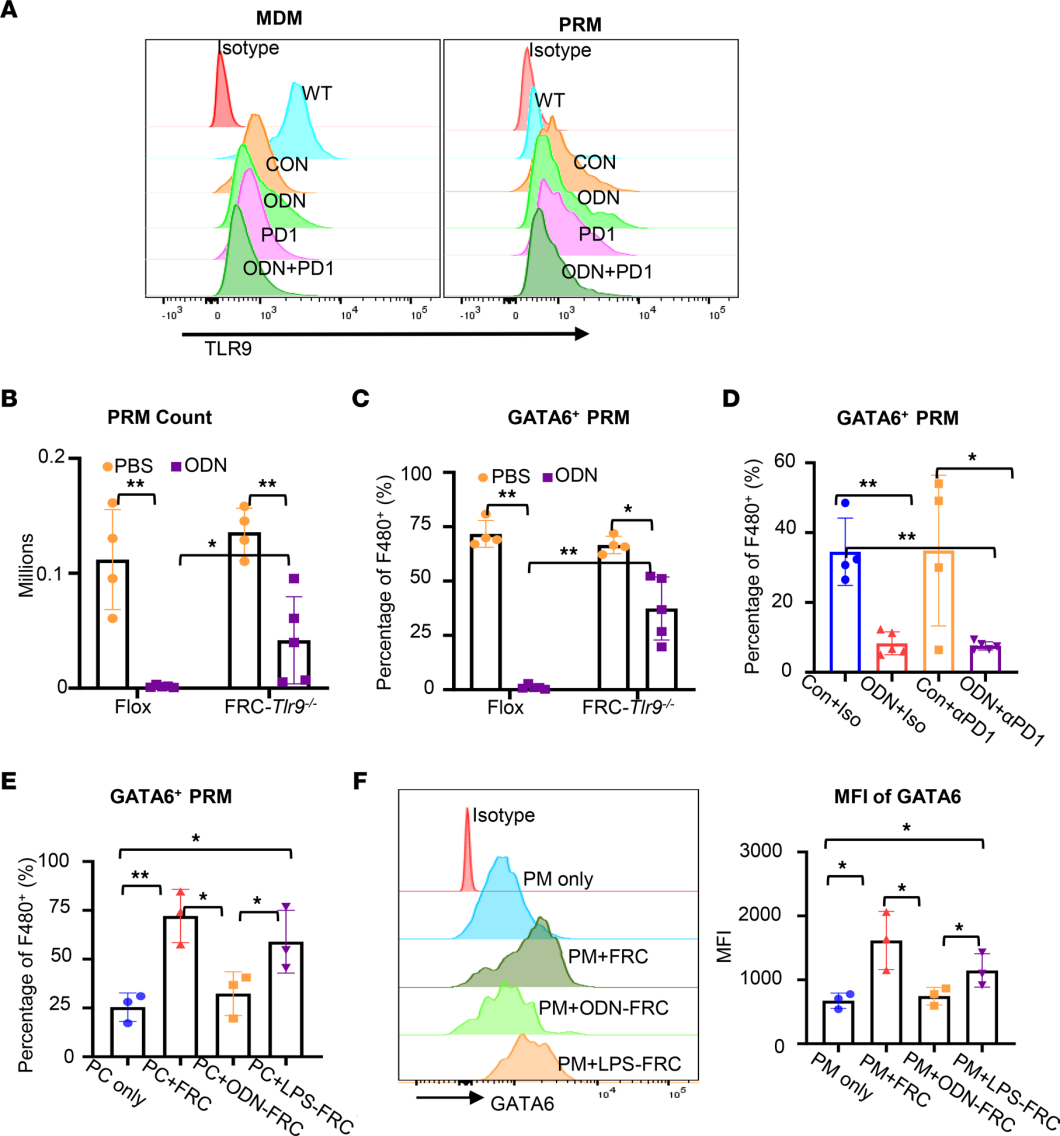
Activation of TLR9 in FRCs induced PRM disappearance via suppression of GATA6 expression in PRMs. (**A**) The expression levels of TLR9 in PRMs and monocyte-derived macrophages (MDMs) in naive WT mice and mice inoculated with MC38 tumor for 3 weeks, with indicated treatments, were analyzed using flow cytometry. (**B** and **C**) Flox and FRC-*Tlr9^–/–^* mice were treated with PBS or ODN1585 (ODN; 5 nmol/mouse, i.p.) for 18 hours. (**B**) The numbers and (**C**) percentages of GATA6^+^ PRMs were assessed using flow cytometry. (**D**) Mice were inoculated with MC38-luciferase-GFP (1 million cells/mouse, i.p.) and received treatments for 3 weeks, as indicated in Figure 1A. The percentage of GATA6^+^ PRMs in peritoneal lavage fluid were analyzed using flow cytometry. (**E** and **F**) Peritoneal cells were isolated from WT mice and cultured with or without control FRCs, ODN1585-preconditioned FRCs (2.5 μM, 18 hours), or LPS-preconditioned FRCs (1 μg/mL, 18 hours) for 18 hours. (**E**) The percentage of GATA6^+^ PRMs in F4/80^+^ macrophages and (**F**) mean fluorescence intensity of GATA6 were analyzed using flow cytometry. (**B–D**) Data are shown as mean ± SD from 2 separate experiments. (**E** and **F**) The experiments were repeated 3 times. Data are shown as mean ± SD from 1 representative experiment. Symbols represent individual mice. Statistical differences were determined using 1-way ANOVA with Tukey’s multiple comparisons test. *P < 0.05, **P < 0.01. Only the significant differences are labeled.

To understand the mechanism underlying TLR9-induced PRM disappearance, we first analyzed TLR9 expression patterns in PRMs. Using flow cytometry on cells in peritoneal washings, we distinguished PRMs (CD11b^hi^F4/80^hi^) from monocyte-derived peritoneal macrophages (CD11b^lo^F4/80^lo^). Monocyte-derived peritoneal macrophages expressed a high level of TLR9 at baseline (Figure 4A). The expression levels of TLR9 in monocyte-derived peritoneal macrophages decreased at week 3 after MC38 tumor inoculation, regardless of treatment (Figure 4A). TLR9 was barely detectable in PRMs at baseline and at week 3 after tumor inoculation (Figure 4A), suggesting that TLR9 in other peritoneal cell types might regulate the retention of PRMs. FRCs in the omentum have been shown to maintain PRM homeostasis (19). We have recently demonstrated that TLR9 regulates peritoneal immunity via regulating chemokine expression in FRCs (17). Therefore, we hypothesized that TLR9 in FRCs is involved in TLR9-induced PRM disappearance. To test this hypothesis, we generated FRC-specific *Tlr9^–/–^* mice and subjected these mice along with Flox control mice to challenge with ODN1585. The total numbers and frequencies of PRMs were significantly higher in the FRC-specific *Tlr9*^–/–^ (FRC-*Tlr9^–/–^*)mice than in Flox mice at 18 hours after ODN1585 challenge (Figure 4, B and C), suggesting that FRC-TLR9 was involved in the induction of PRM disappearance.

A previous study demonstrated that FRCs regulate the expression of GATA6 in PRMs (19). GATA6, the lineage-defining transcription factor of PRMs, controls the phenotype, survival, and peritoneal retention of PRMs (8, 20, 23, 24). As expected, we found that the percentage of GATA6^+^ PRMs markedly decreased at week 3 after MC38 tumor inoculation in mice treated with ODN1585 only or ODN+anti–PD-1 compared with mice that received control isotype antibody and anti–PD-1 (Figure 4D). To test if FRC-TLR9 regulates GATA6 expression in PRMs, we isolated peritoneal cells from WT mice and cocultured these cells with or without control FRCs, LPS-preconditioned FRCs, or ODN1585-preconditioned FRCs for 18 hours. Only 25% of the PRMs were GATA6^+^ when cultured without FRCs (Figure 4E). The GATA6^+^ PRMs dramatically increased to 70% in the cocultures with control FRCs or LPS-preconditioned FRCs (non-TLR9 TLR-preconditioned control) (Figure 4E). However, when cocultured with ODN1585-preconditioned FRCs, the GATA6^+^ PRMs were substantially reduced (Figure 4E). Similar results in the mean fluorescence intensity for GATA6 expression were observed in cocultures of ODN1585-preconditioned FRCs compared with control FRCs or LPS-preconditioned FRCs (Figure 4F). Together, these data suggest that activation of TLR9 in FRCs induces PRM disappearance via suppression of GATA6 expression in PRMs.

### Activation of TLR9 negatively regulates retinoid metabolism in FRCs.

**Figure 5:**
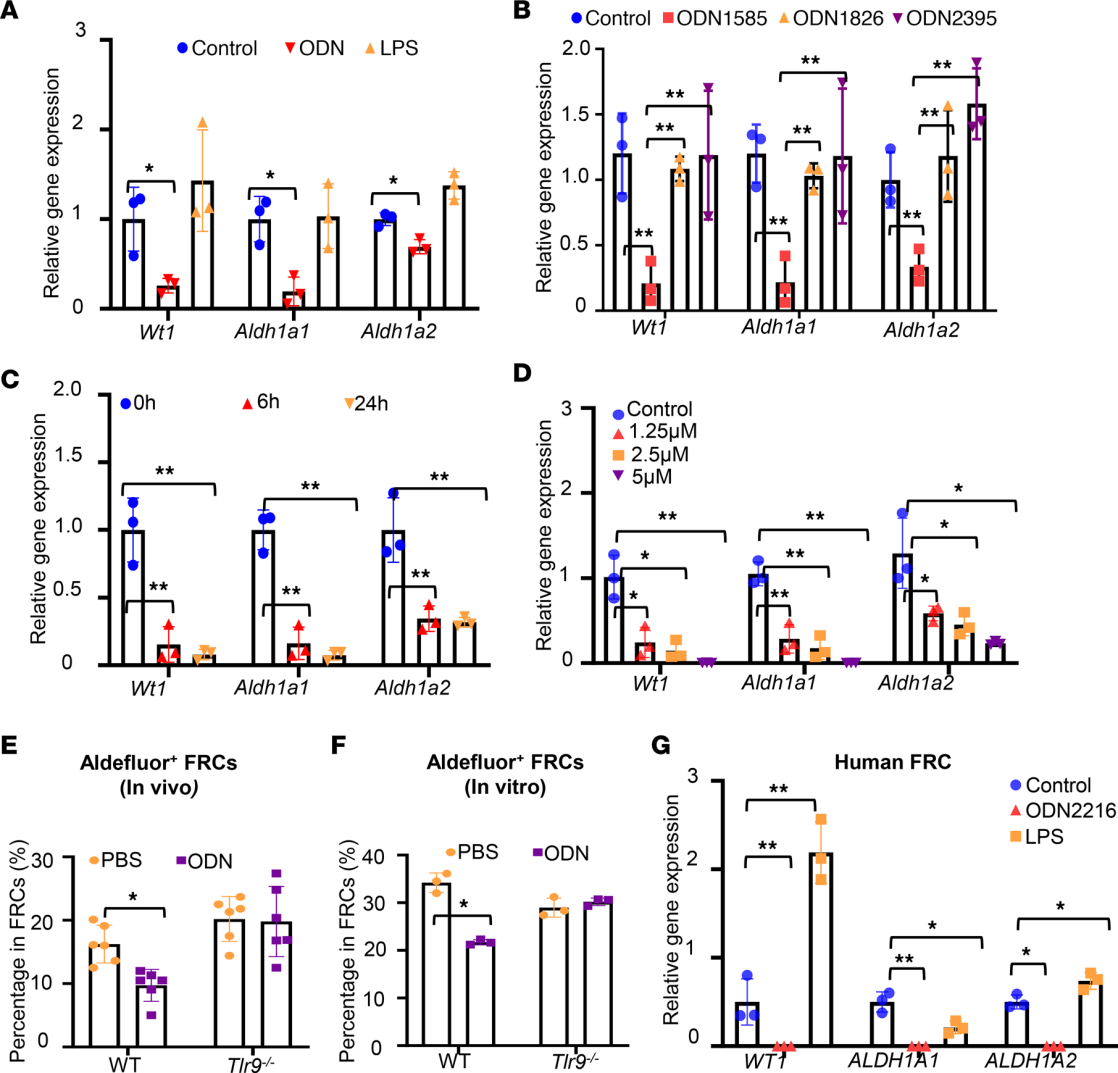
Activation of TLR9 negatively regulates retinoid metabolism in FRCs. (**A–D**) Abundance of *Wt1*, *Aldh1a1*, and *Aldh1a2* transcripts in mouse FRCs (**A**) with control, ODN1585 (2.5 nM),or LPS (1 μg/mL) treatment for 18 hours; (**B**) with ODN1585 (2.5 nM), ODN1826 (2.5 nM), or ODN2395 (2.5 nM) treatment for 18 hours; (**C**) with ODN1585 (2.5 nM) treatment for indicated time periods; or (**D**) with indicated concentrations of ODN1585 treatment for 18 hours. (**E**) Percentage of ALDEFLUOR^+^ FRC in vivo. WT and *Tlr9^–/–^* mice were treated with PBS or ODN1585 (5 nmol/mouse, i.p.) for 18 hours. ALDEFLUOR^+^ FRCs in the omentum and mesenteric adipose tissue were analyzed using flow cytometry. (**F**) Percentage of ALDEFLUOR^+^ FRCs in vitro. WT and *Tlr9^–/–^* FRCs were treated with PBS or ODN1585 (2.5 nM) for 18 hours. ALDEFLUOR^+^ FRCs were analyzed using flow cytometry. (**G**) Abundance of *WT1*, *ALDH1A1*, and *ALDH1A2* transcripts in human FRCs with control, ODN2261 (2.5 nM), or LPS (1 μg/mL) treatment for 18 hours. (**A–D**, **F**, and **G**) The experiments were repeated 3 times. Data are shown as mean ± SD from 1 representative experiment. (**E**) Data are shown as mean ± SD from 2 separate experiments. Symbols represent individual mice. Statistical differences were determined using 2-way ANOVA with Tukey’s multiple comparisons test. *P < 0.05, **P < 0.01. Only the significant differences are labeled.

Retinoid acid derived from FRCs has been shown to drive GATA6 expression in PRMs (19). To test if TLR9 regulates retinoid metabolism in FRCs, mRNA levels of retinoid metabolism-related genes (RMGs; *Wt1*, *Aldh1a1*, and *Aldh1a2*) were assessed in mouse FRCs after ODN1585 or control treatment (PBS or LPS as a non-TLR9 control). Of note, RMG expression levels dropped substantially in FRCs with ODN1585 treatment compared with that after control treatments (Figure 5A). The suppression of RMG expression was only observed in FRCs treated with ODN1585 but not in FRCs treated with ODN1826 or ODN2395 (Figure 5B). The suppression of RMG expression was observed at both 6 and 24 hours after ODN1585 treatment (Figure 5C). Furthermore, ODN1585 suppressed RMG expression in FRCs in a dose-dependent manner (Figure 5D).

The aldehyde dehydrogenases (ALDHs), including ALDH1a1 and ALDH1a2, are the rate-limiting enzymes of the last step of retinoic acid synthesis (retinaldehyde to retinoic acid) (25). To determine the effect of TLR9 activation on the activities of ALDH, WT and *Tlr9^–/–^* mice were treated with PBS or ODN1585 for 18 hours, and ALDH activity in FRCs isolated from FALCs was analyzed using an ALDEFLUOR assay kit. Compared with PBS controls, treatment with ODN1585 decreased the percentage of ALDEFLUOR^+^ FRCs in WT but not in *Tlr9^–/–^* mice (Figure 5E). Consistent with the in vivo findings, in vitro treatment of cultured FRCs with ODN1585 also decreased the ALDEFLUOR^+^ FRCs only in WT FRCs but not in *Tlr9^–/–^*-FRCs (Figure 5F).

To determine whether TLR9 signaling also regulates RMG expression in human FRCs, we isolated FRCs from lipoaspirates of human adipose tissue and expanded them ex vivo. We found that the levels of RMGs dropped substantially in human FRCs with ODN2216 treatment but not with LPS treatment (Figure 5G). Together, these data indicate that the class A TLR9 agonist suppressed retinoic acid metabolism in FRCs.

### Blocking TLR9 in FRCs diminished the benefit of ODN1585 in anti–PD-1 therapy for peritoneal metastases.

**Figure 6:**
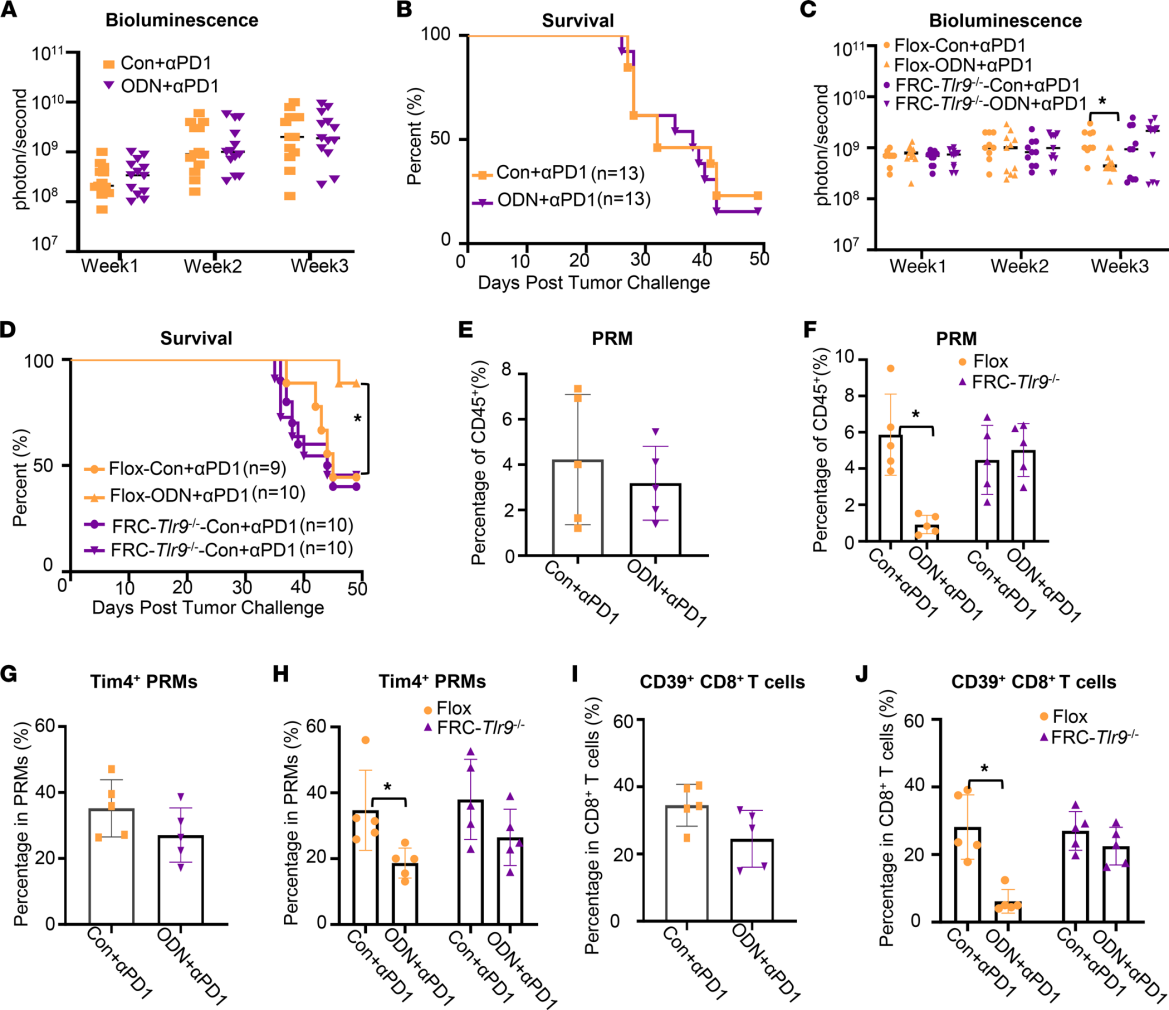
Blocking TLR9 in FRCs diminished the benefit of ODN1585 in anti–PD-1 therapy for peritoneal metastases. Mice were inoculated with MC38-luciferase-GFP (1 million cells/mouse, i.p.) as indicated in Figure 1A. (**A**) Bioluminescence measured as photons per second in global *Tlr9^–/–^* mice at the indicated times after peritoneal tumor challenge. (**B**) Kaplan-Meier survival curve for global *Tlr9^–/–^* mice with indicated treatment for 49 days. (**C**) Bioluminescence measured at indicated time points after tumor inoculation in Flox, and FRC-*Tlr9^–/–^* mice treated as indicated. (**D**) Kaplan-Meier survival curve for Flox and FRC- *Tlr9^–/–^* mice with indicated treatment for 49 days. (**E** and **F**) The percentage of PRMs in CD45^+^ cells in (**E**) global *Tlr9^–/–^* mice and (**F**) Flox and FRC-*Tlr9^–/–^* mice at week3 after MC38 inoculation. (**G** and **H**) The percentage of (**G**) Tim4^+^ PRMs in global *Tlr9^–/–^* mice and (**H**) Flox and FRC-*Tlr9^–/–^* mice at week3 after MC38 inoculation. (**I** and **J**) The percentage of (**I**) CD39^+^CD8^+^ T cells in global *Tlr9^–/–^* mice and (**J**) Flox and FRC-*Tlr9^–/–^* mice at week 3 after MC38 inoculation. Data are from 2 separate experiments. Symbols represent individual mice. Statistical differences were determined using (**A** and **C**) 2-way ANOVA with Tukey’s multiple comparisons test, (**B** and **D**) log-rank test, (**E**, **G**, and **I**) unpaired, 2-tailed Student’s *t* tests, and (**F**, **H**, and **J**) 1-way ANOVA with Tukey’s multiple comparisons test. *P < 0.05. Only the significant differences are labeled.

To test if FRC-TLR9 is required for the benefit of ODN1585 in anti–PD-1 treatment for peritoneal metastases, we inoculated global *Tlr9^–/–^* mice, FRC-*Tlr9^–/–^* mice, and the Flox control mice with MC38-luciferase-GFP cells and treated these mice with anti–PD-1 or ODN+anti–PD-1 every 3 days, starting 1 week after tumor inoculation (Figure 1A). As expected, the tumor burden and 7-week mortality were similar in global *Tlr9^–/–^* mice and FRC-*Tlr9^–/–^* mice between anti–PD-1 and ODN+anti–PD-1 treatments (Figure 6, A–D). There were no associated differences in the percentage of PRMs, Tim-4^+^ PRMs, and CD39^+^CD8^+^ T cells in global *Tlr9^–/–^* mice and FRC-*Tlr9^–/–^* mice between anti–PD-1 and ODN+anti–PD-1 treatments (Figure 6, E–J). In contrast, the tumor burden; the 7-week-mortality rate; and the percentage of PRMs, Tim-4^+^ PRMs, and CD39^+^CD8^+^ T cells in Flox mice that received ODN+anti–PD-1 treatment decreased markedly compared with that in mice that received anti–PD-1 treatment alone (Figure 6, C, D, F, H, and J). These data indicate that TLR9 in FRCs is responsible for the beneficial effect of adding ODN1585 to anti–PD-1 to treat peritoneal metastases.

## Discussion

In this study, we found that the addition of a class A TLR9 agonist (ODN1585) enhanced the effectiveness of anti–PD-1 ICB in reducing colorectal peritoneal metastases. In parallel, the activation of the TLR9 signaling in FRCs induced PRM disappearance from the peritoneal cavity. Mechanistically, activation of TLR9 in FRC blocked the crosstalk between FRCs and PRMs via suppression of retinol metabolism in FRCs, which is essential for driving GATA6 expression in Tim4^+^ PRMs and, thus, enhances CD8^+^ T cell antitumor immunity.

Colorectal peritoneal metastases present or develop in about 25% of patients with colorectal cancers (1). The prognosis of patients with colorectal peritoneal metastases is poor, and there is currently no established standard treatment (6). A previous study reported that 50% of patients with colorectal peritoneal metastasis are suitable for immunotherapy (26). Therefore, first-line immunotherapy could have a central role in managing patients with peritoneal recurrence from microsatellite-instable/mismatch repair–deficient colorectal cancer and even be an adjunct to surgery (27). Although ICB immunotherapies have shown promising therapeutic efficacy in various malignant diseases (28), peritoneal metastases are associated with a low response rate to ICB and poor clinical outcomes after ICB therapy (5).

Studies that aim to develop strategies to improve the response rate of ICB therapy have shown that combining TLR9 agonists with anti–PD-1 ICB therapy enhances anti–PD-1 treatment in mouse models of pancreatic cancer (15) and head and neck squamous cell carcinoma (16). Consistent with these studies, we show here that the addition of a class A TLR9 agonist (ODN1585) enhanced the antitumor responses of anti–PD-1 therapy in peritoneal metastases derived from microsatellite-instable MC38 and microsatellite-stable CT26 colorectal carcinoma.

Previous studies have shown that treatment with TLR9 agonists enhances anti–PD-1 therapy via altering the tumor immune microenvironment, particularly CD8^+^ T cell immunity (15, 16). In line with these findings, we also found that combining the class A TLR9 agonist and anti–PD-1 therapy enhanced CD8^+^ T cell antitumor immunity. However, we have previously shown that T cells in the peritoneal cavity barely express TLR9 (17), suggesting the TLR9 agonist does not directly act on T cells to regulate their functions. Here, we found that PRMs barely expressed TLR9 at baseline and after peritoneal tumor inoculation, as opposed to monocyte-derived macrophages. This led us to examine the role of peritoneal stromal cells in the TLR9-driven responses. Stromal cell–derived retinoid acid drives GATA6 expression in PRM. GATA6 expression in PRM is critical for PRM retention and survival in the peritoneal cavity. (19). We have previously shown that activation of TLR9 signaling in FRCs, a subpopulation of stromal cells, suppresses chemokine production in FRCs and, thus, suppresses immune cell recruitment during sepsis (17). By showing that TLR9 regulates FRC retinoid metabolism, we extend understanding of the mechanisms by which FRC-TLR9/retinoids and PRM/GATA6 crosstalk regulate peritoneal immunology.

PRMs, a unique population of macrophages, maintain peritoneal homeostasis and provide immune surveillance in the peritoneal cavities of mice (8, 20, 23, 24) and humans (29). Xia et al. have shown that resident macrophages expressing Tim4 (a receptor for phosphatidylserine) promoted tumor growth in a mouse model of ovarian cancer with peritoneal metastasis (4). Inhibiting mitophagy in macrophages results in a loss of PRMs and, thus, prevents ovarian cancer metastasis via enhancing T cell–mediated antitumor immunity (4). Furthermore, a recent study has shown that PRMs express high levels of Tim4, which are associated with reduced levels of CD8^+^ T cells in pleural effusions and peritoneal ascites in patients with lung cancer (5). Mechanistic studies reveal that Tim4^+^ PRMs sequester phosphatidylserine in cytotoxic CD8^+^ T cells, thus impairing CD8^+^ T cell proliferation (5). In line with these studies, we have found that the class A TLR9 agonist substantially reduces Tim4^+^ PRM numbers, which are associated with a substantial decrease in CD39^+^CD8^+^ exhausted T cells in the peritoneal cavity.

PRMs are originally derived from embryonic progenitors and undergo self-renewal (30). During homeostasis, PRMs are nonadherent in peritoneal fluid (22). In response to infection and inflammation, PRMs rapidly disappear from the peritoneal fluid. This phenomenon is called MDR. Recent studies on the mechanisms of MDR have revealed that PRMs become adherent to the mesothelium, driving peritoneal immune cell aggregation and clot formation for bacterial clearance (13, 22). PRMs then subsequently undergo pyroptotic cell death (13). These data indicate that PRM adherence, aggregation, and cell death are involved in MDR in immediate responses to infection or inflammation. Whether similar responses are also involved in the early disappearance of PRM in response to class A ODN is uncertain and will require further research.

With the advances in lineage-tracing studies, emerging evidence demonstrates that monocyte-derived macrophages progressively replace native PRMs during inflammation (31). PRM levels are reported to return to baseline at 1 week after infection (13). However, we have observed here that PRMs remain at substantially low levels, even at 3 weeks after tumor inoculation in mice treated with ODN1585, suggesting that ODN1585 impairs the differentiation of monocyte-derived macrophages to PRMs. GATA6 is the lineage-defining transcription factor controlling cell fate decisions, migration, and biological functions of PRMs (8, 20, 23, 24, 32). Specific deletion of GATA6 in myeloid cells dramatically reduces the number of PRMs and suppresses PRM adhesion (22), migration (8), and proliferation (23). Stromal cells in the omentum are responsible for retinoid metabolism in the peritoneal cavity. Retinoid acid derived from stromal cells sustains GATA6 expression in PRM during homeostasis (19, 20). Here, we have shown that class A TLR9 agonists suppress the expression level of retinol metabolism–related genes and the activities of the rate-limiting enzymes of retinoic acid synthesis in FRCs. Therefore, it is conceivable that class A TLR9 agonist may negatively regulate the PRM replenishment via suppression of retinol metabolism in FRCs, which is essential in driving GATA6 expression in monocyte-derived macrophages for differentiation to PRMs. According to our knowledge, this is the first report that ODN1585 suppresses the expression of retinol metabolism–related genes in cells. The mechanisms of how class A TLR9 agonists suppress retinol metabolism in FRCs are unknown. Further studies are needed to understand the underlying mechanisms of class A ODN–induced suppression of retinol metabolism in FRCs.

In summary, we have provided compelling evidence that activation of TLR9 in FRCs led to the disappearance of Tim4^+^ PRMs, which enhanced antitumor immunity and responses to anti–PD-1 therapy in peritoneal metastasis. These findings warrant further study of combined treatment using class A TLR9 agonists and ICB as a potentially novel immunotherapeutic strategy for peritoneal metastases. Furthermore, our studies suggest that crosstalk signaling between PRM and FRC may be targeted to improve the efficacy of ICB therapy for peritoneal metastases.

## Methods

### Reagents.

LPS-EB (tlrl-eblps), CpG ODN 1585 (tlrl-1585), CpG ODN 1826 (tlrl-1826), CpG ODN 2395 (tlrl-2395), CpG ODN 2216 (tlrl-2216), CpG ODN 2336 (tlrl-2336) were purchased from InvivoGen.

### Mice.

WT C57BL/6J mice, BALB/cByJ mice, and *Myd88^–/–^* mice were purchased from The Jackson Laboratory. *Tlr9^–/–^* and *Tlr9^loxp/loxp^* (Flox) mice (33) on the C57BL/6J background were from Mark J. Shlomchik’s laboratory (Department of immunology, University of Pittsburgh). *Ccl19*-cre mice (34) were obtained from Burkhard Ludwig (Institute of Immunobiology, Kantonsspital St. Gallen, St. Gallen, Switzerland). FRC-*Tlr9^–/–^* mice were generated with the Cre-loxP technique and were identified by PCR-based genotyping, using multiple primer pairs, as described previously (17). These animals were bred in our facilities. All mice were maintained under pathogen-free conditions. Mice were randomly assigned to different experimental or control groups at between 6 and 8 weeks of age. Upon arrival, all mice were acclimated in or animal facility for 3 or more days prior to any experiments.

### Isolation and ex vivo expansion of mouse FRCs.

Mice were sacrificed and blood was extracted by cardiac puncture. Omentum were collected. Mesenteric adipose tissue was carefully excised from the small intestine, large intestine, and cecum using scissors without rupture of intestines. Lymph nodes were removed. Pancreas was kept untouched. Omentum and mesentery adipose tissue were minced in RPMI 1640 Medium (Gibco) containing 2% FBS (Biotechne), 60 μg/mL Liberase TL (MilliporeSigma), and 250 μg/mL DNase I (MilliporeSigma). Minced adipose tissue was digested at 37°C for 25 minutes of shaking. Digestion was stopped by adding 4 times the volume of RPMI 1640 with 10% FBS. Digested tissue was filtered through a 70 μM sterile filter and then centrifuged for 5 minutes at 250*g*. Supernatant was discarded, and cells were resuspended in MesenCult Expansion medium (STEMCELL Technologies Inc.). Cells were cultured at 37°C with 5% CO_2_ and used for in vitro experiments after 7 days.

### RNA extraction, cDNA synthesis, and quantitative PCR.

Total RNA of cultured cells was extracted using the RNeasy Mini Extraction Kit (QIAGEN) per the manufacturer’s instructions. RNA quality and concentration were measured using BioTek Synergy Mx and TAKE 3 plate system (both from Agilent). Two-step, real-time reverse-transcription PCR was performed as previously described (35), with forward and reverse primer pairs prevalidated and specific for indicated target genes (Supplemental Table 1). All samples were assayed in duplicate and normalized to actin mRNA abundance.

### Peritoneal macrophage and FRC coculture assay.

Peritoneal cells were isolated from peritoneal lavage fluid. FRCs were isolated and cultured as above. FRCs were plated in 6-well flat-bottom plates at a concentration of 100,000 FRCs/well and cultured for 18 hours before coculture with peritoneal cells. For preconditioning, FRCs were treated with ODN1585 (2.5 μM) or LPS (1 μg/mL) for 18 hours. FRCs were washed with PBS for 3 times before coculture with peritoneal cells (500,000 cells/well). Cells were cultured in complete RPMI 1640 supplemented with 10 ng/mL MCSF (R&D Systems, 415-ML-005/CF) for 18 hours. Cells were deattached by trypsin and a cell scraper before flow cytometry analysis.

### ALDEFLUOR assay.

FALC cells were isolated as above. For in vivo experiments, once a single-cell suspension (1 mouse) was obtained, cells were resuspended in 1 mL ALDEFLUOR assay buffer (STEMCELL Technologies Inc.) in FACS tubes. For in vitro experiments, 50,000 cells/mL FRCs were used. Reagent was prepared according to ALDEFLUOR kit instructions (STEMCELL Technologies Inc.). Each sample had a quench tube and a test tube. 5 μL DEAB buffer was aliquoted into quench tubes. 5 μL activated ALDEFLUOR reagent was then added to 1 mL single-cell suspension in a test tube, as soon as possible. Cells were mixed by pipetting, and 0.5 mL of the mixture was transferred to quench tubes immediately. Samples were incubated at 37°C for 20 minutes. Following incubation, all tubes were centrifuged for 5 minutes at 250*g*, and supernatant was discarded. Surface staining was performed as above before flow cytometry analysis.

### Flow cytometry.

Cells were blocked for Fc receptors with anti-mouse CD16/32 (BD Bioscience) for 5 minutes and then were stained with fluorochrome-conjugated antibody (Supplemental Table 2) for 30 minutes, at 4°C in the dark. For intracellular staining, cells were fixed and permeabilized using the Foxp3 Transcription Factor Staining Buffer Set (eBioscience), followed by intracellular staining with antibodies (Supplemental Table 2) for 30 minutes at 4°C in the dark. Flow cytometry data were acquired using a LSRII Flow Cytometer (BD) and LSRFortessa Flow Cytometer (BD) with FASCDiva Software (version 8.0.1, BD Pharmingen) and analyzed with Flowjo software (version 10).

### Culture of human FRCs.

Human adipose tissue–derived stromal cells were obtained from the adipose stem cell center at the Department of Plastic Surgery at the University of Pittsburgh. Human adipose tissue–derived stromal cells were cultured and expanded in MesenCult Expansion full media (STEMCELL Technologies Inc.) at 37°C with 5% CO_2_ for 7 days. The purity of FRCs (CD45^–^CD31^–^PDPN^+^) was assessed using flow cytometry.

### Tumor inoculations.

Mice were randomly assigned to different experimental or control groups at between 6 and 8 weeks of age. All mice were maintained under pathogen-free conditions. For MC38 tumor models, WT Tlr9^–/–^, Flox, and FRC-Tlr9^–/–^ mice with C57BL/6J background were injected with one million MC38-luciferase-GFP cells i.p. For CT26 tumor models, WT BALB/cByJ mice were administered with one million CT26-luciferase-GFP cells i.p. IgG2a isotype antibody (10 mg/kg, Bio X Cell), anti–PD-1 (10 mg/kg, Bio X Cell), ODN1585 (5 nmol/mouse, InvivoGen), or anti–PD-1+ODN were given i.p. every 3 days start at 1 week after tumor cell inoculations.

### Tumor cell culture, luciferase transfection, and bioluminescence imaging.

The murine cancer cell line of MC38 colon carcinoma (Kerafast) and murine colorectal carcinoma cell line CT26.WT (ATCC) were cultured in DMEM (Hyclone supplemented with 10% FBS) and 1% penicillin/streptomycin (Thermo Fisher Scientific) at 37°C in a 5% CO_2_ incubator (Thermo Fisher Scientific). MC38 and CT26 tumor cells expressing GFP and firefly luciferase (FLuc) genes, respectively, were generated using FLuc-F2AGFP-IRES-Puro Lentivirus (Biosettia) and then selected with hygromycin (Thermo Fisher Scientific). For imaging, mice were anesthetized with inhaled isoflurane followed by i.p. injection of potassium luciferin (300 mg/kg; Gold Biotechnology) for 10 minutes. Mice were imaged using the Optical imaging system IVIS Lumina II (PerkinElmer), according to the manufacturer’s instructions. Analysis of resultant data was performed using LIVING IMAGE software (PerkinElmer). Regions of interest were manually selected and quantified for average photon flux (photons/second/cm^2^/steradian) (36).

### Statistics.

Statistical analysis was performed with Prism9 (GraphPad Software). Unpaired, 2-tailed Student’s *t* tests were performed to compare 2 groups. One-way ANOVA or 2-way ANOVA with Tukey’s multiple comparisons tests were performed to compare 3 or more groups, as indicated in the figure legends. *P* values of less than 0.05 were considered statistically significant. All values are presented as mean ± SD. As for survival analysis, statistical differences were determined using the log-rank test.

### Study approval.

All animal studies were approved by the Institutional Animal Care and Use Committees of the University of Pittsburgh and the Animal Care and Use Committee of The Ohio State University. Experiments were performed in adherence to the NIH Guidelines.

## Author contributions

MD and TRB conceived the project. MD, TRB, and HH supervised the study and designed experiments. MD, TJ, TRB, and HH drafted and edited the manuscript. TJ and HZ performed experiments and analyzed the data. YL, PJ, and HL performed experiments. TJ and HZ contributed equally to this study. TJ initiated this study and established the hypothesis of the study and, therefore, is in the first position in the author list. MD and TRB contributed equally to study supervision. MD initiated and led this study and, therefore, is in the last position in the author list.

## Supplementary Material

Supplemental data

## Version 1 10/24/2022

Electronic publication
