# Supplementary material for: Intraperitoneal injection of class A TLR9 agonist enhances anti–PD-1 immunotherapy in colorectal peritoneal metastases
Source: JCI Insight. 2022 Oct 24;7(20):e160063. doi: 10.1172/jci.insight.160063 (PMC9714777; doi:10.1172/jci.insight.160063)
Supplement: Supplemental data [file jciinsight-7-160063-s149.pdf]

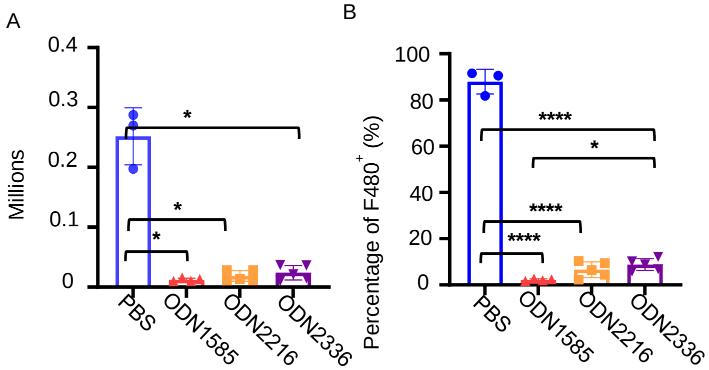

Supplemental Figure 1. Class A TLR9 agonists induced PRM disappearance. WT mice were treated with PBS (100ul, i.p.), ODN1585 (5nmol/mouse i.p.), ODN2216 (5nmol/mouse i.p.), or ODN2336 (5nmol/mouse i.p.) for 18 hours. PRM counts (A) and the percentage of PRM in total F4/80$^+$ cells (B) are shown. Data are shown as mean ± SD from 2 separate experiments. Symbols represent individual mice. Statistical differences were determined using one way ANOVA with Turkey's multiple group comparison test. *p<0.05;****p<0.0001. Only the significant differences are labeled.

Supplemental Table 1 PCR primers

Name	Sequence/description	
Ms_Aldh1a1	Forward Primer	ATACTTGTCGGATTTAGGAGGCT
	Reverse Primer	GGGCCTATCTTCCAAATGAACA
Ms_Aldh1a2	Forward Primer	CAGAGAGTGGGAGAGTGTTCC
	Reverse Primer	CACACAGAACCAAGAGAGAAGG
Ms_WT1	Forward Primer	GAGAGCCAGCCTACCATCC
	Reverse Primer	GGGTCCTCGTGTTTGAAGGAA
Ms_GAPDH	forward primer	AACTTTGGCATTGTGGAAGG
	reverse primer	ACACATTGGGGGTAGGAACA
H_GAPDH	Forward Primer	GGAGCGAGATCCCTCCAAAAT
	Reverse Primer	GGCTGTTGTCATACTTCTCATGG
H_Aldh1a1	Forward Primer	GCCGGAGGAAATGTACCAGAC
	Reverse Primer	CCCCTTGAAGGTAGGGCAG
Hs_ALDH1A2_1_SG QuantiTect Primer Assay	(NM_170697, NM_003888, NM_001206897, NM_170696)	
	GeneGlobe ID - QT00066045	
Hs_WT1_1_SG QuantiTect Primer Assay	(NM_000378, NM_001198552, NM_024425)	
	GeneGlobe ID - QT00059003	

Supplemental Table 2 flow antibody for surface staining

antibody	clone	company
FRC staining		
BUV395 Rat Anti-Mouse CD45	30-F11	BD Biosciences
BUV395 Rat Anti-Mouse CD31	390	BD Biosciences
PE-Cy™7 Rat Anti-Mouse CD31	390	BD Biosciences
Brilliant Violet 421™ anti-mouse CD140a	APA5	BioLegend
APC anti-mouse Podoplanin	8.1.1	BioLegend
PE anti-mouse CD55 (DAF)	RIKO-3	BioLegend
PerCP/Cyanine5.5 anti-mouse CD9	MZ3	BioLegend
Immune cells staining		
PE/Cyanine7 anti-mouse/human CD11b	M1/70	BioLegend
APC/Cyanine7 anti-mouse/human CD11b	M1/70	BioLegend
PerCP/Cy5.5-anti-mouse CD11b	M1/70	BioLegend
PE/Cyanine7 anti-mouse I-A/I-E （MHC II）	M5/114.15.2	BioLegend
Brilliant Violet 605™ anti-mouse F4/80	BM8	BioLegend
Brilliant Violet 421 anti-mouse F4/80	BM8	BioLegend
Alexa Fluor® 700 anti-mouse CD11c	N418	BioLegend
PE-Cy-7 Rat Anti-mouse CD45	30-F11	BD Biosciences
BUV395 Rat Anti-mouse Ly-6G	1A8	BD Biosciences
Alexa Fluor 700 Rat Anti-mouse CD3	17A2	BioLegend
PerCP/Cy5.5-anti-mouse CD4	GK1.5	BioLegend
V450 anti-mouse CD8a	53-6.7	eBioscience
APC anti-mouse CD39	Duha59	Biolegend
PE anti-mouse CD8a	53-6.7	Biolegend
AF700 anti-mouse CD4	RM4-5	Biolegend
APC anti-mouse FOXP3	FJK-16s	Biolegend
FITC anti-mouse T-bet	4B10	Biolegend
Brilliant Violet 421 anti-mouse CD279(PD1)	29F.1A12	Biolegend
PE/Cyanine7 anti-mouse IFN-γ	XMG1.2	Biolegend
Alexa Fluor647 anti-mouse Tim-4	RMT4-54	BioLegend
Fixable Viability Dye eFluor™ 780		Biolegend
Intracellular staining		
PE-anti-mouse GATA-6	D61E4	Cell Signaling
PE Mouse Anti-Mouse CD289 (TLR9)	J15A7	BD Biosciences
